# Diagnostic Accuracy of Right Bronchial Infiltration on Chest X-rays in Diagnosing COVID-19 Patients in the Early Stage of the Disease

**DOI:** 10.7759/cureus.23351

**Published:** 2022-03-21

**Authors:** Asad Ullah Wasim, Rukhsana Khan, Muhammad Sheharyar Khan, Zillehuma Mustehsan, Muhammad Wasim Khan

**Affiliations:** 1 Department of Medicine, Fazaia Medical College, Islamabad, PAK; 2 Division of Clinical and Translational Research, Larkin Community Hospital, South Miami, USA; 3 Department of Community Medicine, Fazaia Medical College, Islamabad, PAK; 4 Department of Internal Medicine, Al Iman General Hospital, Riyadh, SAU

**Keywords:** accuracy, infiltration, chest x-ray, reticular opacities, covid-19

## Abstract

Purpose

SARS-CoV-2 has been a diagnostic challenge for healthcare setups worldwide since 2019 due to its proximity to a myriad of pathological processes. Although reverse transcription - polymerase chain reaction (RT-PCR) and high-resolution computed tomography (HRCT) have helped in the diagnosis of the disease, they are not as widely available as chest X-rays. This study aims to investigate the diagnostic accuracy of right bronchial infiltration in chest X-ray in diagnosing COVID-19.

Material and methods

This was a validation study conducted in a single center in Riyadh, Saudi Arabia. A total of 114 patients were enrolled according to the selection criteria of the study. Consent was waived off on the condition of confidentiality maintenance as per the ethical review board. X-rays of suspected patients were viewed and analyzed by two blinded consultant radiologists. Patients were followed for their RT-PCR reports. Data were entered and analyzed in SPSS Statistics v.23.0 (IBM Corp., Armonk, USA).

Results

Among the 114 patients, the mean age was 46.2±17.3 years and 85 (74.6%) were males. The total number of COVID-19-positive patients were 82 (71.9%) while the patients presenting with right bronchial infiltration (RBI) were 94 (82.5%). RBI was significantly associated with the presence and absence of COVID-19 on PCR (p<0.001) and the presence of comorbidities (p<0.001). The sensitivity, specificity, positive predictive value, negative predictive value, and accuracy of the sign were 84.04%, 85.00%, 96.34%, 53.12%, and 84.21%, respectively.

Conclusions

RBI can be used as a diagnostic sign in X-rays for early identification of COVID-19 positive patients. This feature can be used in the triage of patients. This would decrease the spread of disease by providing early time to intervene to isolate patients.

## Introduction

Healthcare systems have been greatly impacted by the coronavirus disease 2019 (COVID-19) worldwide. Belonging to the family of respiratory viruses, this highly transmissible virus first appeared in Wuhan, China and was named severe acute respiratory syndrome coronavirus 2 (SARS-CoV-2) [1, 2]. The disease may present asymptomatically but may also progress to severe symptoms. Cough, anosmia, dysgeusia and shortness of breath are some of the primary complaints a symptomatic patient has. However, the disease may progress to a widespread immunological response leading to cardiac, renal, and respiratory complications. All these complications ultimately translate into functional impairment of the patient [3].

Multiple laboratories and radiological diagnostic modalities have been invented and made available for the prompt diagnosis of the disease [4]. From simple chest radiography to high-resolution computed tomography (HRCT) scans in radiology, and from serological diagnosis to the widely accepted reverse transcription polymerase chain reaction (RT-PCR), each modality has its own benefits and drawbacks [5-11]. Currently, RT-PCR has been recommended as the detection of SARS-CoV-2, while HRCT for severity assessment. RT-PCR has its own limitation such as the wait time, high expense, shortage of kits and low in-patient sensitivity ranging from 62.9-71.9% [12]. HRCT has been considered as the gold standard in diagnosing SARS-CoV-2 as there are incidences where COVID-19 RT-PCR has come false positive. A variety of features are appreciable on HRCT which help us delve into the nature of the disease [13]. It may begin early with reticular changes in the lungs but soon this progress to ground-glass opacities, crazy paving pattern, lobar and segmental involvement, mediastinal lymph node swelling and extra-pulmonary manifestations such as pericardial effusion [14]. However, the only drawback HRCT has is that the imaging modality is not as widely available in developing countries and depending on it for the final diagnosis leads to a lot of burden on healthcare [15, 16]. Every single scan takes a certain amount of time and it cannot be as robust and quick as a chest X-ray (CXR).

In the abnormal CXRs of covid patients, consolidation opacities are the most common finding seen, followed by reticular interstitial thickening, ground-glass opacities. Pulmonary nodules and pleural effusion are also appreciated in some cases. Most of the patients show bilateral lung affection with peripheral distribution and lower zone affection [8, 12]. However, these changes are late changes generally seen in chest X-rays. Prior to these changes, COVID-19 disease can be possibly detected if early changes in the X-rays are timely appreciated. Among these early changes is the presence of right bronchial infiltration (RBI). The aim of this study is to study the diagnostic accuracy of RBI in chest X-rays, which may aid in the early detection of COVID-19 in patients.

## Materials and methods

This is a single-centered, retrospective study carried out at Al-Iman General Hospital, Riyadh. The study population consisted of those patients who were admitted to the Emergency Department and were clinically suspected of having COVID-19 infection. The sampling technique used was non-probability consecutive sampling. 

Selection criteria of patients 

All patients presenting with the suspicion who underwent Chest X-rays in the emergency department were sought. The patients underwent PCR swabs immediately after getting their X-rays done. All those patients who were probable cases of COVID-19 according to hospital policy were then included in the study. Their reports and films were analyzed retrospectively by two consultant radiologists who were blinded from each other’s assessment.

The inclusion criteria included suspected COVID-19 patients with typical symptoms (cough, shortness of breath, documented fever, and body aches), oxygen saturation below 94%, and COVID-19 suspicion based on recent exposure to COVID-19-positive patients. 

The exclusion criteria consisted of patients having non-viral pneumonia, tuberculosis, asthma, chronic obstructive pulmonary disease, and pulmonary edema. The exclusion criteria included those X-rays that were completely whited out, showing that patients had progressed far into the disease and were too late to appear in the ED department. These patients were excluded based on systemic examination and patient history which was available retrospectively in notes. 

Basis, findings and analysis

According to the normal human anatomy, the trachea divides at the point of the carina into two primary bronchi, which then enter the corresponding lung at its hilar (hilum) region. The physiological anatomy of the right primary bronchi is different from the left such that anything that is aspirated has a very high probability of entering the right primary bronchus. First of all, it is larger in diameter, therefore, making it easier for any physical substance with a larger size or greater quantity to enter in comparison to the left primary bronchus. Secondly, the right primary bronchus is held in such a way that it is relatively more vertical from the region of the carina [17]

**Figure 1:**
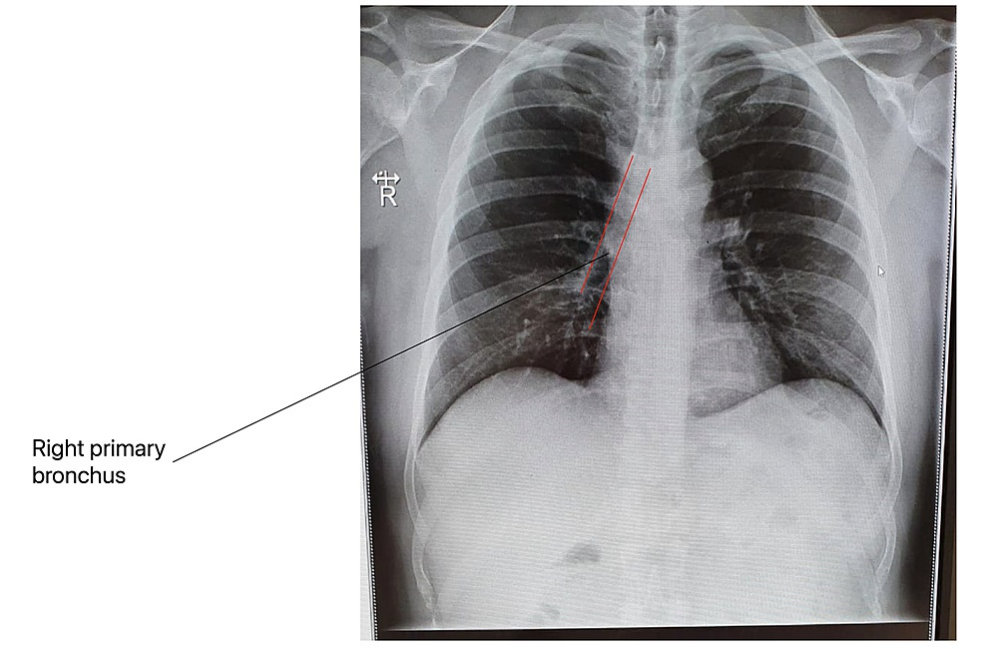
Normal chest X-ray

Moving forward in the right primary bronchus, it is divided into three lobar bronchi, which direct towards the upper, middle, and lower lobes respectively. The lower lobar bronchus specifically is again more vertical, steep, and directs straight down into the lowermost lobe of the lung [17] (Figure 1).

**Figure 2:**
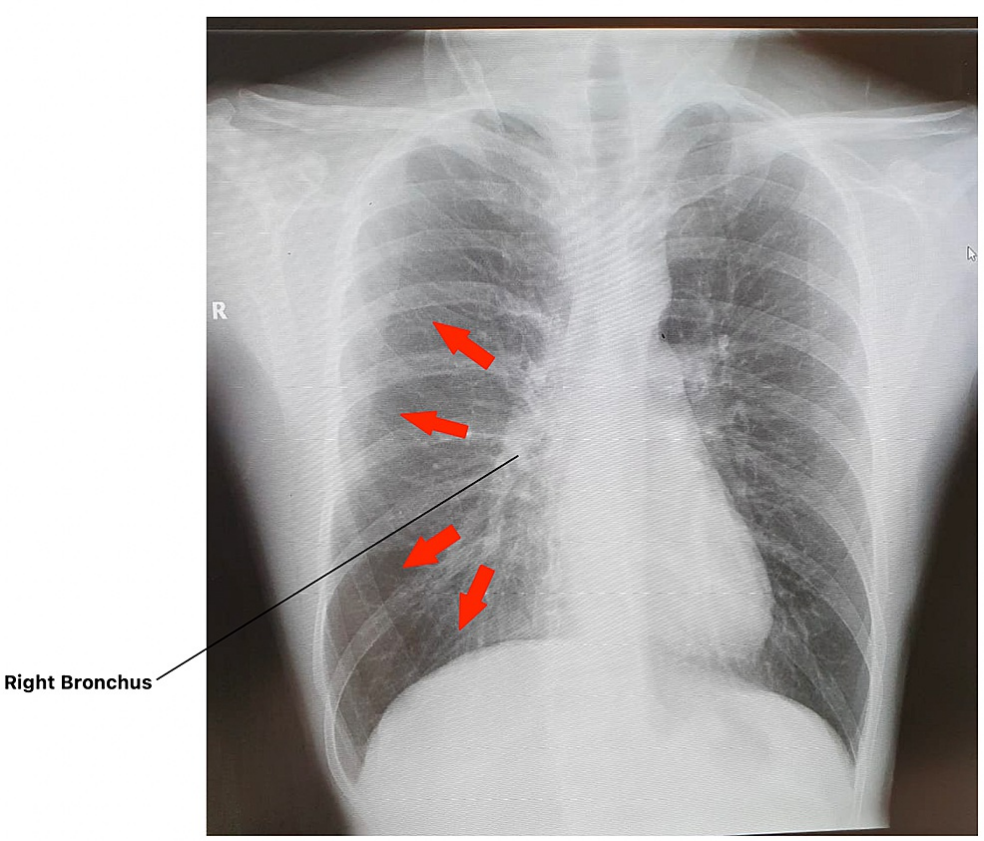
Pathway of infiltration

By understanding this anatomy, we were able to appreciate that all of these patients that came in within a few days of infection were showing infiltrates in their lung CXRs, very significantly inside the right one in the form of white interstitial reticular opacities. We named this the right bronchial infiltration (RBI) during our study. And the most important point to remember here is that these infiltrates were not spreading in an irregular manner; in fact, they followed the pathway from the beginning of the hilar region to right lower lobe in a manner where the actual radiological infiltrate mass was decreasing from the top, down to the right lower lobe (Figure 2).

**Figure 3:**
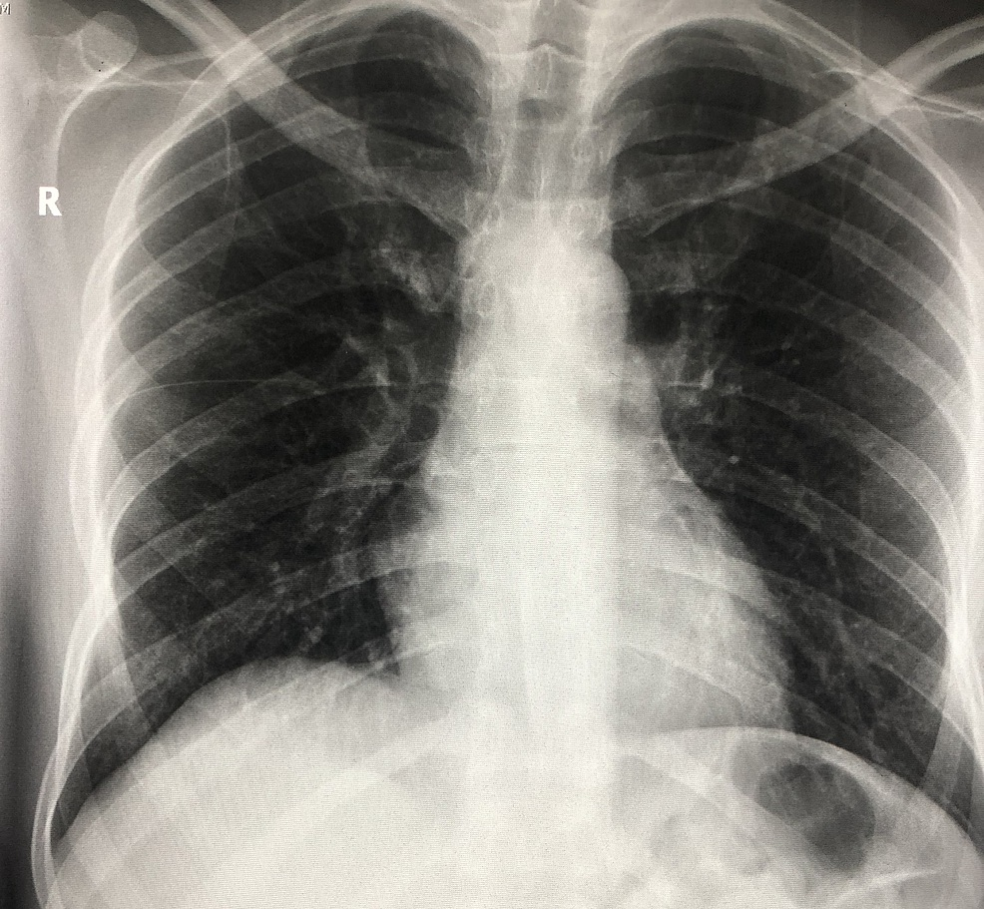
Normal chest X-ray - no signs of bronchial Infiltrates

**Figure 4:**
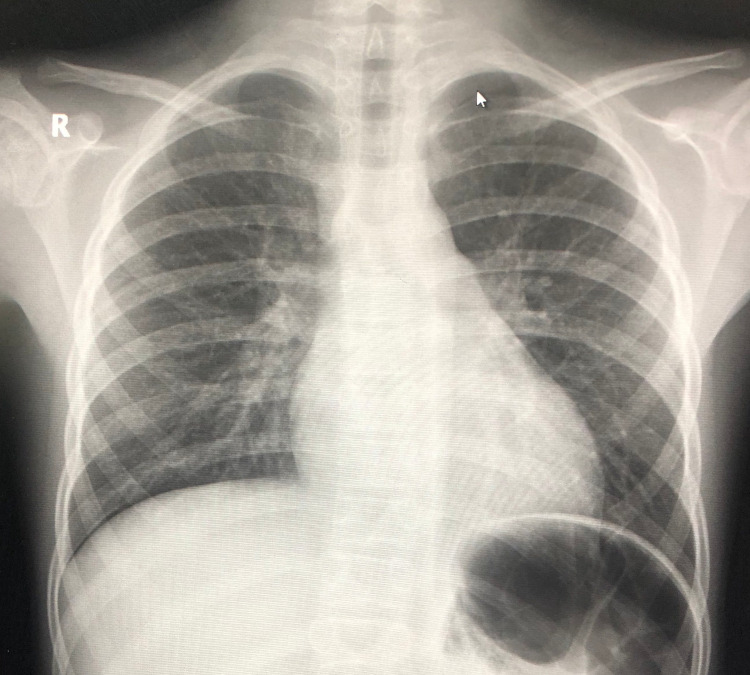
One to two days (expected) since first contact with SARS-CoV-2 SARS-CoV-2: severe acute respiratory syndrome coronavirus 2

**Figure 5:**
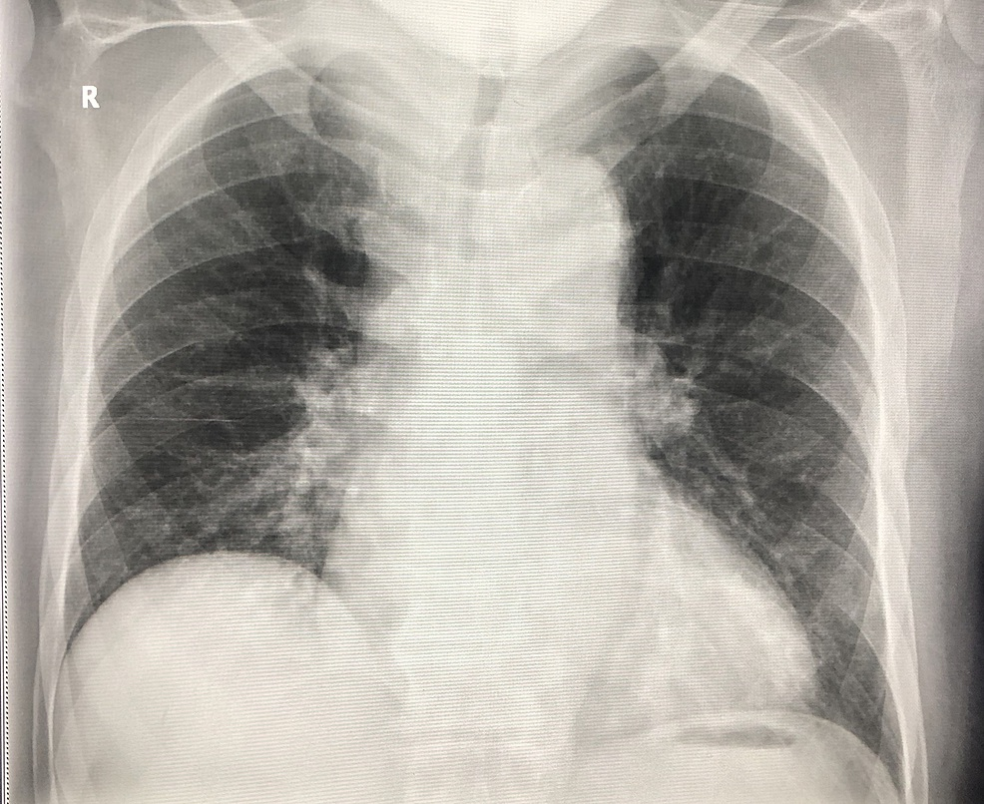
Five to seven days (expected) since first contact - increased viral load

**Figure 6:**
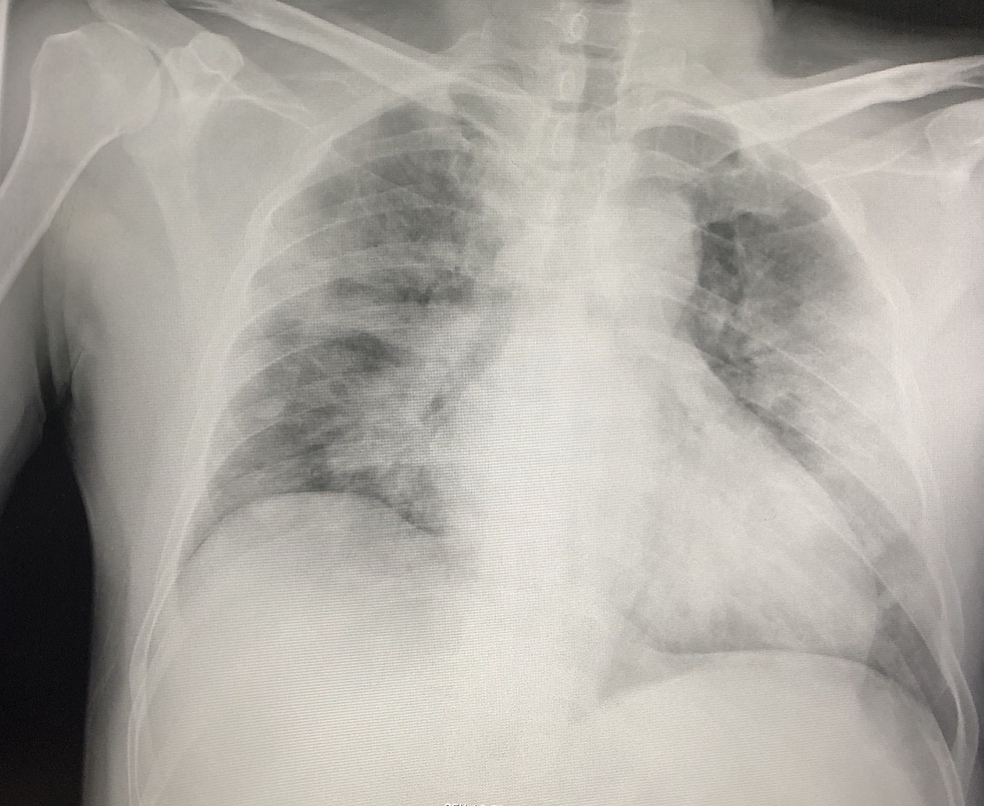
Two to three weeks since the first contact

As the disease progressed, the infiltrates seen on radiological X-rays were discovered to radiate outward from this channel, producing worsening in the form of massive infiltrates that caused consolidated pneumonia. This sometimes further leads to acute respiratory distress syndrome. Figures 3-6 depict the predicted period following first contact with COVID-19 and how this progression appears on a chest X-ray.

The CXRs were reviewed and evaluated by two blinded experts: consultant radiologists (of more than 25 years of experience). They observed the X-rays thoroughly without any knowledge of the PCR reports. They separated the patient IDs with normal chest X-rays and those with positive RBI signs by displaying them in a table format, which was later modified by adding the COVID-19 results, comorbidities, etc. To ensure an assessment solely on radiology, patients with COVID-19 negative results were later excluded from the table along with those patients with no sign of bronchial infiltrations.

Equipment, software and image acquisitions

We were able to store and analyze all the X-rays using the picture archiving and communication system (PACS). All the CXRs were taken using the same portable X-ray machine unit (Siemens MOBILETT XP digital, Siemens Healthcare GmbH, Erlangen, Germany). Each X-ray has its own patient identification number (ID) through which we were able to correlate the X-rays with the clinical symptoms of the patients on an integrated web-based hospital system called OASIS (OASIS, Jeddah, Saudi Arabia). The same patient ID can be conveniently used on both PACS and OASIS.

Data entry tool

The data were entered in a self-structured proforma by the principal investigator of this study. Two separate proforma were provided to the consultant radiologists which were supplemented to the original proforma. Demographic variables include age, gender and ethnicity were recorded. Symptoms of the patients were also recorded along with comorbidities.

Statistical analysis

The data were entered and analyzed in SPSS Statistics IBM v.23.0 (IBM Corp., Armonk, USA). Categorical variables were expressed as frequency and percentages, while continuous variables were described using mean ± standard deviations. The outcome variable, frequency of RBI, was presented as a percentage of the total number of patients who presented with a positive COVID-19 PCR result. Data were then stratified according to COVID-19, age≥50, gender and presence of comorbidities. Fischer's exact test was used to analyze the data. A p-value of below 0.05 was considered statistically significant. The validity of the diagnostic sign was measured using the tests of diagnostic accuracy. Sensitivity, specificity, positive predictive value, negative predictive value and accuracy of the test were calculated.

## Results

This research comprised a total of 114 patients. The patients' average age was 46.217.3 years (11-85 years). Males made up 85 (74.6%) of the total number of participants. As seen in Table 1, the patients presenting to the hospital were of various nationalities, with the majority of them being Saudi and Indian.

**Table 1:** Nationalities of the participants

Nationality	Frequency	Percent
Saudi	29	25.4%
Indian	20	17.5%
Yemeni	13	11.4%
Sudanese	9	7.9%
Pakistani	7	6.1%
Bangladeshi	6	5.3%
Filipino	6	5.3%
Egyptian	3	2.6%
Nigerian	3	2.6%
Ethiopian	2	1.8%
Jordanian	2	1.8%
Syrian	2	1.8%
Nepali	2	1.8%
Moroccan	1	0.9%
South African	1	0.9%
Unknown	8	7.0%
Total	114	100.0

**Figure 7:**
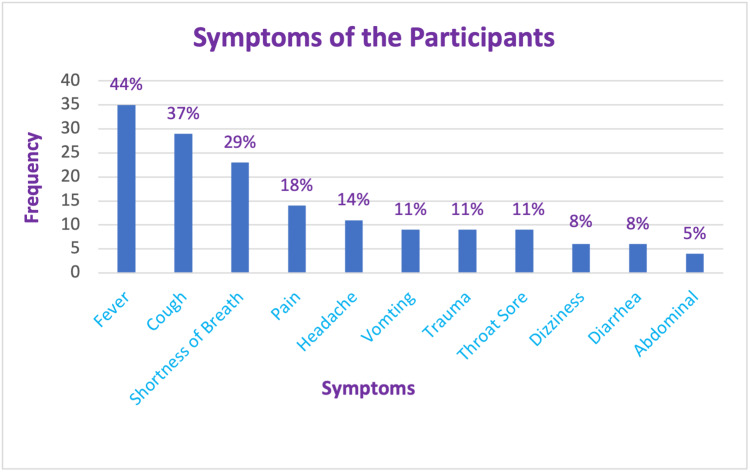
Symptoms of the patients

Symptomatic presentation of the patients is shown in Figure 7.

The total number of COVID-19-positive patients were 82 (71.9%) in this study while the patients presenting with RBI were 94 (82.5%). Thirty (26.3%) patients presented with comorbidities. Table 2 shows the cross-tabulation between RBI and COVID-19, gender, age groups, comorbidities and age ≥50.

**Table 2:** Cross tabulation between RBI and COVID-19, gender, comorbidities and age ≥50 RBI: right bronchial infiltration

		RBI in X-ray		P-value
		Positive	Negative	
COVID-19	Positive	79 (84.0%)	3 (15.0%)	<0.001
	Negative	15 (16.0%)	17 (85.0%)	
Gender	Male	67 (71.3%)	18 (90.0%)	0.065
	Female	27 (28.7%)	2 (10.0%)	
Comorbid	Yes	79 (84.0%)	15 (75.0%)	<0.001
	No	15 (16.0%)	5 (25.0%)	
Age ≥50	Yes	45 (47.9%)	12 (60.0%)	0.230
	No	49 (52.1%)	8 (40.0%)	

Table 3 shows the accuracy, sensitivity, specificity, positive predictive value and negative predictive value of RBI in the diagnosis of COVID-19 patients. 

**Table 3:** Diagnostic accuracy of right bronchial infiltrates CI: confidence interval

Statistics	Value	95% CI
Sensitivity	84.04%	75.05% to 90.78%
Specificity	85.00%	62.11% to 96.79%
Positive Predictive Value	96.34%	90.24% to 98.68%
Negative Predictive Value	53.12%	40.76% to 65.12%
Accuracy	84.21%	76.20% to 90.37%

## Discussion

COVID-19 has been a diagnostic challenge to clinicians all over the world. This is majorly due to the overlapping symptoms the disease has with other diseases such as other viral pneumonia, non-viral pneumonia, pulmonary edema, chronic obstructive pulmonary disease, asthma etc. In developing countries specifically, not only is overlapping with other conditions a cause of missed diagnosis but also the unavailability of resources such as serial X-rays and CT scans [2]. Although COVID-19 PCR testing revolutionized the diagnosis of the disease, its availability is yet another question due to high costs, delayed results and unacceptability among the masses [4].

A person is likely to get an X-ray once and the concept of serial X-rays is relatively not accepted in countries like Pakistan. A study conducted in Pakistan on 150 patients reported that there was a retention of 134 (89.3%) patients after the initial chest X-ray [6]. Adding further, most patients who present with dyspnea are likely to undergo a chest X-ray prior to a CT scan [7]. Given the prevailing health facilities and conditions, it is imperative to look for early changes in X-rays in COVID-19 disease so prompt diagnosis can be made. Early diagnosis and detection of cases allow for the use of early isolation techniques, which reduces disease spread. 

According to previous studies, early changes in X-rays begin with reticular involvement of the lungs [8]. The sensitivity and specificity of chest X-rays have been reported to be less compared to CT scans (69% vs 97%), but in some studies, it has been reported to be equivalent [9,16]. It has been widely accepted that the diagnostic accuracy of X-rays varies with the acumen of the radiologist. 

This study presents a new feature for the radiologists to appreciate called the RBI. Reticular opacities if recognized early in the right bronchial tree can lead to an early diagnosis as this study provides high sensitivity (84.04%) and specificity (85.00%) rates of the feature. When compared to patients with other diagnoses, the presence of RBI was significantly higher in COVID-19 patients (p<0.001). Although, there is minimal research conducted on reticular opacities presenting early in right bronchus in COVID-19 pneumonia, early involvement of the right lower lobe is an indication that the right lung gets affected early. This feature was reported in a recent study mentioning that the posterior basal segment of the right lower lobe was relatively more affected than the left (89.7% vs 64.2%). Adding further, apical (67.9% vs 46.2%), anterior (59.7% vs 48.7%) and posterior (85.9% vs 66.7%) of the upper lobe were more affected in the right side compared to the left [15].

A study conducted in India also reported similar findings, bilateral involvement was reported in 90.2% of the cases but with cases of unilateral involvement majority of them had right lung involvement (7.8%), while only 2% had unilateral left lung involvement. The same study also reported that the right upper lobe and right lower lobe were comparatively more involved when compared to the left side [18]. This perspective was also affirmed by a study conducted in China, which reported that the right lower lobe and upper lobes had higher CT severity scores than the left-sided respective lobes [19]. Conclusively, it is fair to say that COVID-19 disease has right-sided predilection. This study proves that right-sided predilection can be appreciated early in form of right-sided bronchial reticular opacities (RBI). 

A study was conducted in Hong Kong which reported accuracy of 89.5 %, sensitivity of 83.3 %, specificity of 90.0 % for a set of 13 radiomic features. However, this study was conducted in the later stages of COVID-19 disease and made no comments on early changes in radiological imaging [20]. Similarly, another study reported specificity and sensitivity of 89% and 44% for crazy paving patterns and consolidations, but this too was a late presentation of the disease [21]. Similarly, another study reported specificity and sensitivity of 53.8% and 67.9% for ground-glass opacities, 65.7% and 35.7% for consolidation and 64.7% and 53.8% for ground-glass opacities and consolidation combined [22]. Compared to the above-mentioned studies, our study reported higher sensitivity and specificity for the occurrence of RBI in chest X-rays while taking COVID-19 PCR as the gold standard. An advantage this study has is of being able to diagnose COVID-19 in the early phase through this suggested chest X-ray feature. There is relatively less literature available that discusses the sensitivity and specificity of specific features of CT scans and chest X-rays, specifically in the early phase of COVID-19 disease. Hence, our study adds value to the current knowledge we have regarding the value of individual and specific features of radiological tests in diagnosing COVID-19.

Limitations

The study focuses on chest X-ray, which in itself has variations according to different setups and compliance of the patients. False positives and false negatives may be caused on chest X-rays due to lack of inspiration, which can lead to the scapula and breast tissue in females projecting into the lung fields. This leads to increased peripheral density and can lead to false diagnoses. Moreover, this study doesn’t compare the COVID-19 positive patient's RBI with other types of pneumonia, hence further studies are warranted to find out if RBI is only specific to COVID pneumonia. The proportion of controls in this study was less which may have affected the results.

## Conclusions

The purpose of this study was to see if the presence of right bronchial infiltration (RBI) in chest X-rays could accurately diagnose COVID-19 in the early stages of the disease. It was observed during our study that RBI was significantly associated with the presence and absence of COVID-19 on PCR (p<0.001), suggesting that it could be employed as a diagnostic indicator in X-rays for early detection of COVID-19 positive patients. This feature can be used to triage patients, which can help to prevent disease transmission by allowing for early isolation of patients.
